# Picky Eating in School-Aged Children: Sociodemographic Determinants and the Associations with Dietary Intake

**DOI:** 10.3390/nu13082518

**Published:** 2021-07-23

**Authors:** Hebah Alawi Kutbi

**Affiliations:** Clinical Nutrition Department, Faculty of Applied Medical Sciences, King Abdulaziz University, P.O. Box 80215, Jeddah 21589, Saudi Arabia; hkutbi@kau.edu.sa

**Keywords:** picky eating, picky eaters, nutrient intake, dietary intake, children, cardiovascular health

## Abstract

Children exhibiting picky eating behavior often demonstrate strong food preferences and rejection of particular foods or food texture, which may lead to limited dietary variety and possibly inadequate or unhealthy diet. Yet, the relationship between picky eating and nutrient intake in school-aged children has not been established previously. This study aimed to investigate the sociodemographic determinants of picky eating and the associations between picky eating and dietary intake in children. Data of 424 healthy Saudi children aged 6–12 years were collected from their mothers. A child’s picky eating habits were captured using a validated questionnaire. Sociodemographic characteristics of the children were assessed. Dietary data, including 24 h dietary recalls and frequency of fruit, vegetable, and milk consumption, were collected by dietetic professionals using phone-administered interviews. Compared to those of normal-weight mothers, children of mothers with obesity had higher odds of being in the highest tertile of picky eating (OR = 1.93; 95% CI 1.02, 3.63). Children exhibiting higher levels of picky eating consumed less fruits (B = −0.03; 95% CI −0.06, −0.01), vegetables (B = −0.05; 95% CI −0.07, −0.02), and protein (B = −0.21; 95% CI −0.33, −0.09), and had higher consumption of trans fatty acid intake (B = 1.10; 95% CI 0.06, 2.15). Children with higher levels of picky eating presented unhealthy dietary behaviors. Future studies are needed to examine the long-term effect of picky eating on cardiovascular health. Dietary behaviors of mothers with obesity must be taken into consideration when designing intervention programs aiming to improve eating behaviors of children.

## 1. Introduction

Picky eating is a common eating behavior among children [1,2,3,4,5,6], often causing considerable stress to parents and caregivers [6]. Children exhibiting picky eating behavior often demonstrate strong food preferences and rejection of particular foods or food texture [7], which may lead to limited dietary variety and possibly inadequate or unhealthy diet [8,9]. This is particularly relevant when the child rejects the consumption of healthy foods as it may raise the concern for not meeting dietary recommendations or replacing healthy foods by unhealthy food choices [8].

As parents become concerned for the child not eating a variety of foods, they may utilize different feeding practices to overcome the child’s pickiness, which may further hinder the development of healthy dietary behaviors. For instance, the current data indicate that mothers of picky eaters tend to use more pressure on the children to eat and less monitoring of their dietary behaviors [10]. As such, parents may allow the child to consume any of the preferred foods to compensate for the limited food intake [10].

The existing evidence highlight the need to identify barriers to healthy food choices and a clear understanding of factors that impede acceptance and consumption of healthy foods [7]. Therefore, a substantial body of literature has examined the relationship between picky eating and food intake among children. A common theme is that picky eaters tend to demonstrate unhealthy dietary behaviors as compared to non-picky eaters; they have lower intake of vegetables and fruits [3,8,10,11,12], meat, and fish [10,12] than non-picky eaters, and higher intakes of savory snacks, sweets, sugary drinks, and French fries [3,10,13]. However, there is no clear evidence that nutrient intakes of picky eaters differ than that of non-picky eaters. Only one study quantified nutrient intakes of preschoolers and reported lower iron and zinc intakes in picky eaters [8]. Additionally, very little is known about health outcomes of picky eating [14]. Some studies investigated the associations of picky eating with children’s growth and weight status and reported inconsistent findings [15,16]. Other studies suggest that picky eaters experience constipation more commonly than non-picky eaters [17,18].

Existing data suggests high prevalence of picky eating among children. For instance, a study conducted among Chinese children reported a prevalence of 59.3% [1]. In Saudi Arabia, the prevalence of picky eaters was estimated as 89.8% [2]. Other studies reported rates of picky eaters ranging from 13 to 47.4% [3,4,5,6]. As such, many studies have been devoted to understanding the factors associated with picky eating. Findings indicated that preschool-aged children of low-income households are more vulnerable to picky eating compared to those of high-income households [10]. The current evidence also suggests that parent–child interaction and the child’s sensory sensitivity are associated with the development of picky eating [19]. Children with greater sensory sensitivity are more prone to become picky eaters. Parental sensitivity and the lower parental structuring may also increase the likelihood of children exhibiting picky eating, which could be due to the limited opportunity for teaching and learning about foods [19].

Due to the high prevalence of picky eating among children and the limited availability of studies that investigate the relationship between picky eating and nutrient intake of children, the present study aimed to explore the sociodemographic determinants of picky eating and to investigate the associations between picky eating and dietary intake in school-aged children. 

## 2. Materials and Methods

### 2.1. Study Sample

For this cross-sectional study, a minimum of 346 children was required, determined by an expected correlation coefficient of 0.15, power of 80%, and α = 0.05 [20]. As mothers in many Arab countries are considered responsible for child feeding [21], data on the children were collected from their mothers. A questionnaire link was circulated using social media (WhatsApp, Twitter, Instagram, and Telegram) along with a statement describing the study objectives and protocol, confidentiality statement, and consent for participation. Mothers of multiple children were instructed to provide data of the youngest child. The questionnaire included questions on sociodemographic characteristics and eating behaviors of the child and asked for a convenient date and time to conduct a telephone interview for 24 h dietary recall(s) collection. 

Five hundred and thirty-nine mothers completed the online questionnaire. Data of healthy Saudi children (6–12 years old) residing in Saudi Arabia were included. The exclusion criteria included participants who did not report dietary data (14.7%, *n* = 79) and children with chronic illnesses or food allergies (6.68%, *n* = 36). A total of 424 children were included in the sample. This study was approved by the Research and Ethics Committee, Faculty of Applied Medical Science at King Abdulaziz University (FAMS-EC-2020-0010).

### 2.2. Measures

#### 2.2.1. Sociodemographic Characteristics

Data were collected regarding the child’s sex and age, region of residence, birth order (first child vs. not), breastfeeding initiation, monthly household income, paternal and maternal education level, maternal age and employment status, and whether the father is involved in child feeding and lives with the child. Mothers were also requested to report their current weight and height, and the body mass index (BMI) was calculated. Maternal weight status was determined based on the World Health Organization (WHO) criteria; maternal weight was defined as normal if the BMI was 18.5–24.9 kg/m^2^, underweight if the BMI was <18.5 kg/m^2^, and overweight or obese if the BMI was 25.0–29.9 or ≥30.0 kg/m^2^, respectively [22].

#### 2.2.2. Picky Eating Scale

The degree of picky eating was assessed using the picky eating subscale from the Child Eating Behavior Questionnaire [23], which has been previously validated among children in Saudi Arabia [24]. The picky eating construct consists of three items: (1) “My child is fussy or picky about what he/she eats”; (2) “My child’s diet consists of only a few foods”; and (3) “My child is unwilling to eat many of the foods that our family eats at mealtime.” Response options for each item ranged from 1 “disagree” to 5 “agree.” The average score of the three items was calculated, wherein a score of 5 indicated the highest level of picky eating. The internal reliability of the construct assessed by Cronbach’s alpha was acceptable (α = 0.70). To evaluate the associations of child characteristics with the degree of picky eating, children were divided into tertiles based on their average picky eating scale score. 

#### 2.2.3. Dietary Assessment 

Dietary data were collected by trained dietetic professionals, and two registered dietitians were responsible for overseeing the collection of dietary data, field validation, and data entry. A phone interview reminder was sent to each mother as a text message a day earlier. During the interview, mothers were requested to estimate the frequency and quantity of the child’s consumption of fruits, vegetables, and milk. The frequency of consumption was reported as “per day,” “per month,” or “per week,” and the average frequency was calculated to represent children’s intake per day. Additionally, a comprehensive education was provided on the serving size of foods and data to disclose when reporting the 24 h dietary recall(s), such as the number of servings, food description, the name of ready-to-eat food manufacturers, etc. Pictures of standardized measurements were sent to mothers to further assist in estimating portion size of food consumed and to ensure correct estimations of food quantity. Another phone interview was scheduled to collect 24 h dietary recall, and mothers were requested to have the child and whoever assists in feeding the child participate in the phone interview. To adjust for within individual variations, three inconsecutive 24 h dietary recalls (one weekend day and two weekdays) were obtained for 168 randomly selected children (39.6%) within two weeks from the first 24 h dietary recall.

The dietary data were analyzed using the dietary analysis software Nutritics, research edition (version 5.6, Dublin, Ireland). The Nutritics software includes official national databases for countries such as Saudi Arabia, and a database of universal Arabic foods. Local standardized food recipes were entered into the software. Density of macronutrients were then calculated as percentages of energy intake, and density of each micronutrient was calculated by dividing the nutrient intake per unit by 1000 kcal.

### 2.3. Statistical Analysis

Data were illustrated as median (interquartile range), mean ± standard deviation, and percentages (frequency). Chi-square test was used to assess differences in child characteristics across the three tertiles of picky eating, whereas Kruskal-Wallis test was used to investigate differences in dietary intakes (2-sided tests). Multinomial regression adjusted for child sex and age was used to examine the independent associations between child characteristics and tertiles of picky eating, wherein tertile I was set as the reference category. The associations between picky eating and dietary intakes were further investigated using linear regression analyses adjusted for child sex and age as covariates. The regression analyses were performed at 95% confidence level. The Statistical Package for Social Sciences (SPSS) was used for data analyses. 

## 3. Results

Nearly half of the children were boys (49.5%, *n* = 210) and lived in households with annual income above 10,000 Saudi riyals (SRs) (54.0%, *n* = 229). Over two-thirds of mothers (75.2%, *n* = 319) and fathers (63.2%, *n* = 268) had a college degree or higher. The majority of children in our sample were living with their mothers and fathers (89.4%, *n* = 379), and about half of the fathers were reported to be participating in child feeding (47.4%, *n* = 201). Thirty-eight percent of the mothers had normal bodyweight (*n* = 161). The prevalence of underweight, overweight, and obesity among mothers was 3.10% (*n* = 13), 36.8% (*n* = 156), and 22.2% (*n* = 94), respectively.

The median picky eating scale score across the total study sample was 3.00 out of a total score of 5.00 (2.33–4.00), with a mean score of 3.07 ± 1.07. Median picky eating scale score among children in tertile I was 2.00 (1.67–2.33), with a mean score of 1.89 ± 0.46. Median and mean picky eating scale scores for children in tertiles II and III were 3.00 (2.67–3.33), 3.00 ± 0.25 and 4.33 (3.67–4.67), 4.25 ± 0.47, respectively. Table 1 describes children’s characteristics stratified by tertiles of picky eating. The univariate analyses did not indicate any significant difference in child characteristics across tertiles of picky eating (*p* > 0.05). However, the multinomial regression analyses adjusted for child sex and age suggests that compared to children of normal-weight mothers, children of mothers with obesity have higher odds of being in the highest tertile of picky eating (OR = 1.93; 95% CI 1.02, 3.63) (Table 2). 

Dietary intakes of children stratified by tertiles of picky eating are shown in Table 3. Significant differences in the frequency of fruit and vegetable consumptions were observed across the picky eater tertile groups; Children in the lowest tertile of picky eating had a mean intake of 1.06 ± 0.95 servings of fruits per day, whereas children in the second and third tertiles consumed 1.04 ± 0.87 and 0.83 ± 0.78 serving of fruits per day, respectively (*p* = 0.01). Children in the lowest tertile, second tertile, and third tertile of picky eating consumed 1.04 ± 0.79, 0.96 ± 0.82, and 0.73 ± 0.81 serving of vegetables per day, respectively (*p* < 0.001). Furthermore, the mean intake of protein was found to be significantly different across the picky eater tertile groups; the mean protein intakes of children in first, second, and third tertiles were 15.9 ± 4.03, 14.9 ± 3.92, and 14.5 ± 4.05 g per 1000 kcal per day, respectively (*p* < 0.01).

Multiple linear regression analyses adjusted for children’s age and sex were conducted to examine the associations of picky eating with dietary intake of children (Table 4). Findings indicated inverse associations between picky eating scale scores and daily intakes of fruit (B = −0.03; 95% CI −0.06 to −0.01), vegetable (B = −0.05; 95% CI −0.07 to −0.02), and protein (B = −0.21; 95% CI −0.33 to −0.09) and a positive association with trans fatty acid intake (B = 1.10; 95% CI 0.06 to 2.15). 

## 4. Discussion

Findings of the present study showed that compared to those children of normal-weight mothers, children of mothers with obesity have higher odds of being in the highest tertile of picky eating. Children exhibiting higher levels of picky eating consumed significantly fewer fruits and vegetables and less protein than those with lower levels, and higher amount of trans fat. 

Dietary behaviors of children can be largely influenced by parental dietary behaviors and practices [15]. Children of parents who are picky eaters themselves tend to exhibit characteristics of picky eating behaviors [25]. Several studies have also observed parent–child commonalities in intakes of healthy and unhealthy foods, particularly for mothers [26,27,28]. In the present study, children of mothers with obesity were found to have higher odds of being in the highest tertile of picky eating than those of normal-weight mothers. This finding indicates that these children are more likely to experience a characteristic that is unique for mothers with obesity. A randomized controlled trial has demonstrated a significant association between obesogenic behaviors of children and mothers; children with obesity were more likely to meet behavioral recommendations for fast food intake, consumption of sugary drinks, and television viewing if their mothers presented lower levels of these behaviors [29]. Although picky eating in mothers has not been evaluated in this study, it is possible that the obese mothers themselves are experiencing picky eating, or that eating behaviors of the obese mothers are linked to their children’s food pickiness. This could be particularly relevant as some studies have shown higher prevalence of obesity among picky eaters than non-picky eaters [25], although other studies reported inconsistent findings [30]. Further studies are needed to reveal how maternal obesity is linked to child’s picky eating. When designing intervention programs aiming to improve eating behaviors of children, children of mothers with obesity must have a particular attention, and maternal dietary behaviors must be considered in order to achieve the desired outcomes. 

Results of this study are aligned with previously reported findings in which children exhibiting picky eating behaviors tend to consume fewer fruits and vegetables and less protein than non-picky eaters [9]. Multiple factors could contribute to limited intake of these foods among children, such as food insecurity [31,32]. In the context of picky eating, some children might be more sensitive to tough texture than non-picky eaters [2], which could explain the limited intake of fruits, vegetables, and meat among children with higher levels of picky eating compared to those with the lower levels. Another possible explanation for the limited intake of protein-rich food sources is that picky eaters might be sensitive to the strong smell of these foods. In a previous study, children with higher levels of picky eating presented higher degrees of disgust and sensory sensitivity to food texture than non-picky eaters [16]. 

Our data indicated that children with higher levels of picky eating tend to consume greater amounts of trans fat. The higher consumption of trans fatty acids might be an indication for high intakes of fried or fast foods, baked goods such as cakes, cookies, crackers, and/or margarines [33,34]. Trans fatty acids are a form of unsaturated fat that can be presented naturally in dairy and meat or formed during hydrogenation of oils to enhance taste and texture of the foods [33,34]. A high consumption of trans fat raises the concern for children’s cardiovascular health and lipid profile, particularly if this behavior was combined with fewer fruit and vegetable intakes. Existing evidence indicates that a high trans fatty acids intake is associated with lower high-density lipoprotein (HDL) and higher low-density lipoprotein (LDL) cholesterol levels, and increases the risk of developing heart diseases and type 2 diabetes [35]. The American Heart Association recommends limiting intakes of trans fatty acid and sugar while increasing fruit and vegetable consumption in order to prevent the development of cardiovascular disease in growing children [36]. As such, food choices of picky eaters must be carefully monitored.

Parents of picky eating children worry the most when the child rejects healthy foods and they may, therefore, pressure the child to eat the rejected food, although this feeding practice has been found to be counterproductive [37,38]. Alternatively, children may benefit from being surrounded by healthy food environment, as recent evidence indicates a negative association between healthy home food environment and picky eating in children [37]. 

The present study is among the first to investigate the association of picky eating with nutrient intake of school-aged children. Picky eating was assessed using a previously validated measurement tool that showed high internal reliability among the sample. However, this study is limited by the convenient sampling of which the participants were recruited. Findings might not be generalizable to children of relatively low socioeconomic status or those of mothers who do not have access to social media platforms.

## 5. Conclusions

The current study indicates that children exhibiting greater picky eating consume fewer vegetables and fruits, less protein, and higher amount of trans fat than children at the lower levels, presenting unhealthy dietary behaviors. The study could be replicated in more diverse populations to explore whether these observations are applicable to children of low socioeconomic status or food insecure families. Future studies may also investigate whether these dietary behaviors will remain into adulthood and examine the long-term effects of picky eating on cardiovascular health. Additionally, our findings highlight the need to explore how the obese mother–child relationship influences a child’s picky eating behaviors. Dietary behaviors of mothers with obesity must be evaluated when designing intervention programs aiming to enhance dietary behaviors of children.

## Figures and Tables

**Table 1:** Differences in characteristics of children among tertiles of picky eating (*n* = 424) ^a^.

Characteristic		Picky Eating			*p*-Value
		Tertile I (*n* = 145)	Tertile II (*n* = 126)	Tertile III (*n* = 153)	
Age in years, *n* (%)	6–7	47 (32.4%)	41 (32.5%)	56 (36.6%)	0.88
	8–9	44 (30.3%)	36 (28.6%)	46 (30.1%)	
	10–12	54 (37.2%)	49 (38.9%)	51 (33.3%)	
Sex, *n* (%)	Boys	67 (46.2%)	56 (44.4%)	87 (56.9%)	0.07
	Girls	78 (53.8%)	70 (55.6%)	66 (43.1%)	
Region of residence, *n* (%)	Western	80 (55.2%)	73 (57.9%)	89 (58.2%)	0.78
	Central	18 (12.4%)	13 (10.3%)	25 (16.3%)	
	Eastern	20 (13.8%)	19 (15.1%)	14 (9.2%)	
	Southern	17 (11.7%)	14 (11.1%)	17 (11.1%)	
	Northern	10 (6.9%)	7 (5.6%)	8 (5.2%)	
Birth order, *n* (%)	Not first child	89 (61.4)	84 (66.7)	90 (58.8)	0.34
	First child	56 (38.6)	42 (33.3)	63 (41.2)	
Maternal age in years, *n* (%)	<31	30 (20.7%)	19 (15.1%)	27 (17.6%)	0.59
	31–40	82 (56.6%)	69 (54.8%)	88 (57.5%)	
	>40	33 (22.8%)	38 (30.2%)	38 (24.8%)	
Maternal weight status, *n* (%)	Normal weight	58 (40.0)	49 (38.9)	54 (35.3)	0.49
	Underweight	6 (4.10)	4 (3.20)	3 (2.00)	
	Overweight	56 (38.6)	46 (36.5)	54 (35.3)	
	Obese	25 (17.2)	27 (21.4)	42 (27.5)	
Maternal education, *n* (%)	≤High school/diploma	43 (29.7)	25 (19.8)	37 (24.2)	0.17
	≥College degree	102 (70.3)	101 (80.2)	116 (75.8)	
Paternal education, *n* (%)	≤High school/diploma	50 (34.5)	48 (38.1)	58 (37.9)	0.78
	≥College degree	95 (65.5)	78 (61.9)	95 (62.1)	
Maternal employment status, *n* (%)	Unemployed	85 (58.6%)	68 (54%)	98 (64.1%)	0.23
	Employed	60 (41.4%)	58 (46%)	55 (35.9%)	
Any breastfeeding during first 6 months of life, *n* (%)	No	19 (13.1)	13 (10.3)	23 (15.0)	0.51
	Yes	126 (86.9)	113 (89.7)	130 (85.0)	
Father lives with the child, *n* (%)	No	9 (6.20)	16 (12.7)	20 (13.1)	0.10
	Yes	136 (93.8)	110 (87.3)	133 (86.9)	
Paternal involvement in child feeding, *n* (%)	No	30 (20.7)	26 (20.6)	42 (27.5)	0.16
	Sometimes	39 (26.9)	35 (27.8)	51 (33.3)	
	Yes	76 (52.4)	65 (51.6)	60 (39.2)	
Monthly household income, *n* (%)	<4000 SR	10 (6.9%)	9 (7.1%)	10 (6.5%)	0.40
	4000–6000 SR	20 (13.8%)	19 (15.1%)	23 (15%)	
	6001–10,000 SR	35 (24.1%)	23 (18.3%)	46 (30.1%)	
	10,001–15,000 SR	39 (26.9%)	30 (23.8%)	39 (25.5%)	
	>15,000 SR	41 (28.3%)	45 (35.7%)	35 (22.9%)	

^a^ Chi-square test was used for categorical variables. Abbreviation: SR, Saudi riyal.

**Table 2:** Adjusted odds ratio for the association between child characteristics and picky eater status ^a^.

Predictor	Adjusted Odds Ratio and 95% Confidence Intervals			
	Tertile II to Reference Category	*p*-Value	Tertile III to Reference Category	*p*-Value
**Sex**				
Boy	0.93 (0.57, 1.50)	0.76	1.52 (0.96, 2.41)	0.07
Girl	1.00 (Reference category)			
**Region of residence**				
Western	1.30 (0.47, 3.64)	0.62	1.45 (0.54, 3.90)	0.47
Central	1.04 (0.31, 3.46)	0.95	1.82 (0.59, 5.58)	0.30
Eastern	1.38 (0.43, 4.37)	0.59	0.86 (0.27, 2.76)	0.80
Southern	1.18 (0.35, 3.97)	0.79	1.27 (0.39, 4.08)	0.69
Northern	1.00 (Reference category)			
**Age**				
6–7 years old	0.97 (0.55, 1.71)	0.90	1.23 (0.71, 2.13)	0.45
8–9	0.90 (0.50, 1.62)	0.72	1.13 (0.64, 1.99)	0.67
10–12	1.00 (Reference category)			
**Birth order**				
Not first child	1.26 (0.76, 2.10)	0.38	0.92 (0.57, 1.49)	0.74
First child	1.00 (Reference category)			
**Any breastfeeding during first 6 months of life**				
No	0.76 (0.36, 1.61)	0.47	1.18 (0.61, 2.28)	0.63
Yes	1.00 (Reference category)			
**Maternal education**				
≤High school/diploma	0.58 (0.33, 1.02)	0.06	0.79 (0.47, 1.33)	0.38
≥College degree	1.00 (Reference category)			
**Paternal education**				
≤High school/diploma	1.17 (0.71, 1.92)	0.54	1.14 (0.71, 1.83)	0.59
≥College degree	1.00 (Reference category)			
**Maternal employment status**				
Unemployed	0.82 (0.51, 1.33)	0.43	1.28 (0.80, 2.05)	0.30
Employed	1.00 (Reference category)			
**Household income**				
<4000	0.82 (0.30, 2.21)	0.69	1.15 (0.43, 3.10)	0.78
4000–6000	0.86 (0.40, 1.84)	0.69	1.35 (0.63, 2.88)	0.44
6001–10,000	0.60 (0.30, 1.17)	0.14	1.56 (0.83, 2.94)	0.17
10,001–15,000	0.70 (0.37, 1.32)	0.27	1.21 (0.64, 2.29)	0.56
>15,000	1.00 (Reference category)			
**Maternal weight status**				
Underweight	0.79 (0.21, 2.97)	0.73	0.55 (0.13, 2.33)	0.42
Overweight	0.96 (0.56, 1.67)	0.89	1.08 (0.63, 1.85)	0.77
Obese	1.27 (0.65, 2.49)	0.49	1.93 (1.02, 3.63) *	0.04
Normal	1.00 (Reference category)			
**Father lives with the child**				
No	0.16 (0.02, 1.40)	0.10	0.10 (0.01, 0.76) *	0.03
Yes	1.00 (Reference category)			
**Paternal involvement in child feeding**				
No	1.02 (0.55, 1.91)	0.94	1.75 (0.98, 3.14)	0.06
Sometimes	1.06 (0.60, 1.87)	0.84	1.66 (0.97, 2.85)	0.07
Yes	1.00 (Reference category)			

^a^ Models are adjusted for child age and sex. * *p* < 0.05.

**Table 3:** Dietary intakes of children stratified by tertiles of picky eating.

Dietary Intake ^a^	Picky Eating			*p*-Value
	Tertile I (*n* = 145)	Tertile II (*n* = 126)	Tertile III (*n* = 153)	
Servings of milk per day	1.23 ± 0.93 1.00 (0.43–2.50)	1.26 ± 0.99 1.00 (0.43–2.50)	1.14 ± 0.84 1.00 (0.43–1.00)	0.64
Servings of fruits per day	1.06 ± 0.95 1.00 (0.43–1.00)	1.04 ± 0.87 1.00 (0.43–1.00)	0.83 ± 0.78 1.00 (0.43–1.00)	0.01 *
Servings of vegetables per day	1.04 ± 0.79 1.00 (0.43–1.00)	0.96 ± 0.82 1.00 (0.43–1.00)	0.73 ± 0.81 0.43 (0.14–1.00)	<0.01 *
Energy (kcal)	1314 ± 385 1205 (1051–1561)	1313 ± 320 1268 (1101–1460)	1310 ± 335 1266 (1094–1467)	0.87
Carbohydrate (%)	48.8 ± 7.50 48.7 (44.0–52.9)	50.3 ± 6.79 50.6 (47.0–54.4)	49.6 ± 8.00 49.3 (44.7–54.2)	0.09
Fat (%)	33.3 ± 6.35 34.1 (29.7–37.6)	32.8 ± 5.98 32.5 (28.3–36.0)	33.9 ± 7.30 34.0 (28.1–38.6)	0.28
Protein (%)	16.0 ± 4.03 15.7 (13.2–18.3)	14.9 ± 3.92 14.4 (12.00–17.1)	14.5 ± 4.05 14.1 (11.7–17.0)	<0.01 *
Sugar (g/1000 kcal)	96.2 ± 72.6 71.6 (47.6–124)	96.4 ± 53.2 87.3 (59.1–128)	94.3 ± 57.2 82.5 (51.8–126)	0.45
Fiber (g/1000 kcal)	14.6 ± 9.70 11.8 (8.13–18.8)	14.7 ± 9.28 12.7 (8.31–19.3)	13.8 ± 9.79 11.5 (8.07–16.0)	0.64
Trans fat (g/1000 kcal)	0.50 ± 0.65 0.28 (0.07–0.69)	1.00 ± 5.82 0.29 (0.07–0.65)	6.56 ± 85.4 0.23 (0.06–0.74)	0.76
Cholesterol (mg/1000 kcal)	296 ± 236 247 (125–388)	235 ± 167 192 (104–323)	260 ± 234 205 (102–325)	0.07
Vitamin D (ug/1000 kcal)	34.8 ± 99.4 5.00 (2.08–21.7)	23.5 ± 43.9 4.74 (2.00–13.7)	25.0 ± 42.2 5.25 (2.17–28.5)	0.87
Calcium (mg/1000 kcal)	818 ± 592 661 (444–1022)	798 ± 463 708 (444–1047)	779 ± 444 699 (452–969)	0.92
Zinc (mg/1000 kcal)	6.85 ± 4.27 5.81 (3.86–8.72)	7.07 ± 4.24 5.92 (4.32–8.72)	6.55 ± 4.25 5.42 (3.72–8.09)	0.42
Iron (mg/1000 kcal)	13.4 ± 9.41 10.7 (7.07–16.7)	12.8 ± 7.26 11.2 (8.19–15.4)	12.2 ± 7.16 10.7 (7.62–14.3)	0.71

^a^ Dietary data are presented as mean ± standard deviation and median (interquartile range). * *p* <0.05.

**Table 4:** Linear regression analyses of picky eating scale scores on dietary intakes of children ^a^.

Dietary Intake	R^2^	B	Standard Error	95% Confidence Intervals	*p*-Value
Servings of milk per day	0.01	−0.03	0.01	−0.05, 0.00	0.06
Servings of fruits per day	0.02	−0.03	0.01	−0.06, −0.01	0.01 *
Servings of vegetables per day	0.03	−0.05	0.01	−0.07, −0.02	<0.01 *
Energy (kcal)	0.01	−2.11	5.27	−12.4, 8.24	0.69
Carbohydrate (%)	0.01	0.09	0.11	−0.14, 0.31	0.44
Fat (%)	0.01	0.11	0.10	−0.09, 0.30	0.29
Protein (%)	0.03	−0.21	0.06	−0.33, −0.09	<0.01 *
Sugar (g/1000 kcal)	0.003	−0.51	0.94	−2.36, 1.33	0.59
Fiber (g/1000 kcal)	0.01	−0.19	0.15	−0.47, 0.10	0.21
Trans fat (g/1000 kcal)	0.02	1.10	0.53	0.06, 2.15	0.04 *
Cholesterol (mg/1000 kcal)	0.02	−6.08	3.28	−12.5, 0.37	0.07
Vitamin D (ug/1000 kcal)	0.02	−0.80	1.02	−2.81, 1.22	0.44
Calcium (mg/1000 kcal)	0.01	−4.08	7.65	−19.1, 11.0	0.60
Zinc (mg/1000 kcal)	0.02	−0.07	0.06	−0.19, 0.06	0.31
Iron (mg/1000 kcal)	0.02	−0.18	0.12	−0.41, 0.06	0.15

^a^ Models are adjusted for sex and age of children. * *p* <0.05.

## Data Availability

The data used to support the findings of this study are available from the corresponding author upon request.
